# Postoperative new-onset atrial fibrillation causing acute embolic occlusion of the superior mesenteric artery: A case report

**DOI:** 10.1097/MD.0000000000025700

**Published:** 2021-04-30

**Authors:** Jinbeom Cho, Dosang Lee

**Affiliations:** Department of Surgery, College of Medicine, The Catholic University of Korea, Seoul, Republic of Korea.

**Keywords:** atrial fibrillation, complication, mesenteric ischemia, surgery

## Abstract

**Rationale:**

Postoperative atrial fibrillation following noncardiac surgery increases mortality, length of hospital stay, and medical expenses; moreover, compared to nonvalvular atrial fibrillation, it poses a similar risk of thromboembolic complications. In this report, we discuss our decision-making process for diagnosis and treatment in case with unexpected postoperative new-onset atrial fibrillation causing acute mesenteric ischemia.

**Patient concerns:**

A 78-year-old male patient received varicose vein stripping and ligation in his right leg. The patient was previously healthy with no known comorbidities. The next day after surgery, he complained of sudden epigastric pain unresponsive to conservative treatment, and new-onset atrial fibrillation was observed on electrocardiography.

**Diagnoses:**

An abdominal computed tomography scan revealed acute embolic occlusion of the superior mesenteric artery

**Interventions:**

Emergent surgical embolectomy was performed successfully. The time to operation from the recognition of abdominal pain was 6 h. Surgical critical care was performed for life-threatening ischemic reperfusion injury.

**Outcomes:**

The patient was discharged from the hospital on the 40^th^ postoperative day.

**Lessons:**

Atypical postoperative abdominal pain unresponsive to conservative treatment should be considered a surgical emergency, and a high level of clinical suspicion for acute mesenteric ischemia is required. Preoperative electrocardiography and postoperative telemetry might be helpful in some asymptomatic patients.

## 1 Introduction

Atrial fibrillation (A-fib) is the most common cardiac arrhythmia in clinical practice. Therefore, many patients receiving noncardiac surgery may have an A-fib before surgery; a recent study reported that 4312 patients had a history of an A-fib among 38,047 patients who underwent noncardiac surgery.^[1]^ Although the incidence of postoperative new-onset A-fib after noncardiac surgery has been reported to be between 0.4 and 3%,^[2–4]^ it might be difficult to manage these patients if they do not present arrhythmia-related symptoms or signs because most patients receiving surgery with a low risk of a major adverse cardiac event (MACE) are not placed under hemodynamic monitoring postoperatively. We recently treated a patient who exhibited an episode of A-fib the day after varicose vein ligation with stripping. This patient had previously been healthy but was diagnosed with acute embolic occlusion of the superior mesenteric artery (SMA), which might be associated with newly developed A-fib.

Here, we report on this rare but critical case to discuss optimal diagnostic and treatment strategies for critical postoperative A-fib. This is the first reported case of new-onset postoperative A-fib causing immediate embolic occlusion of the SMA.

## 2 Case presentation

**Figure 1:**
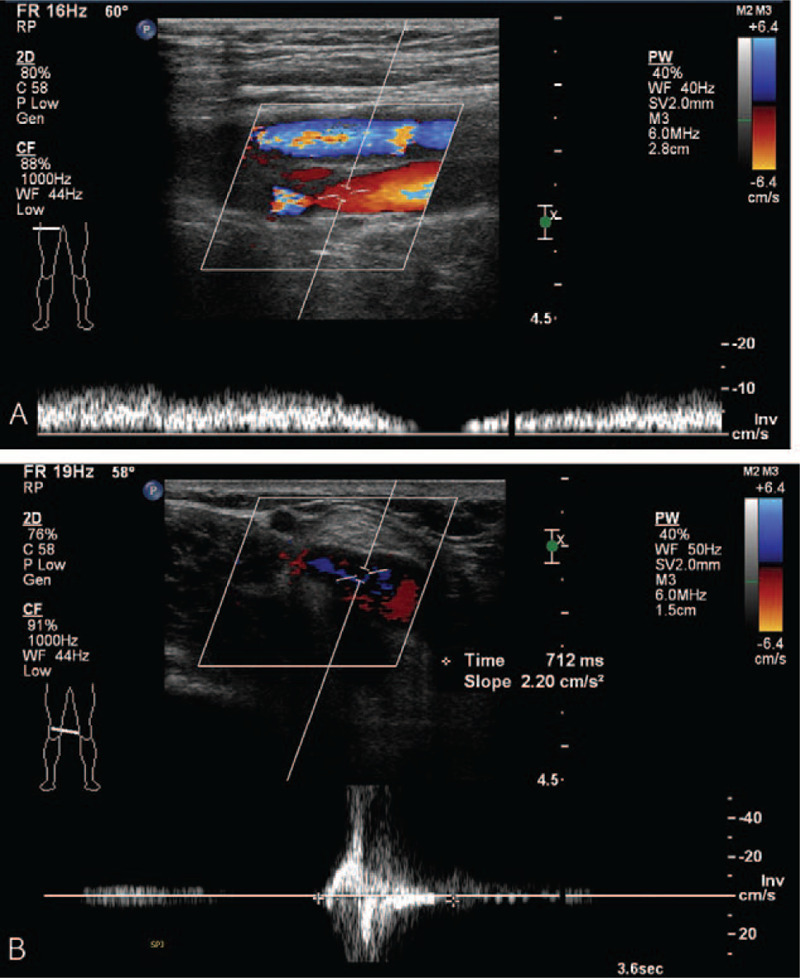
(A) & (B) Duplex sonographic findings of the venous system in the right leg.

**Figure 2:**
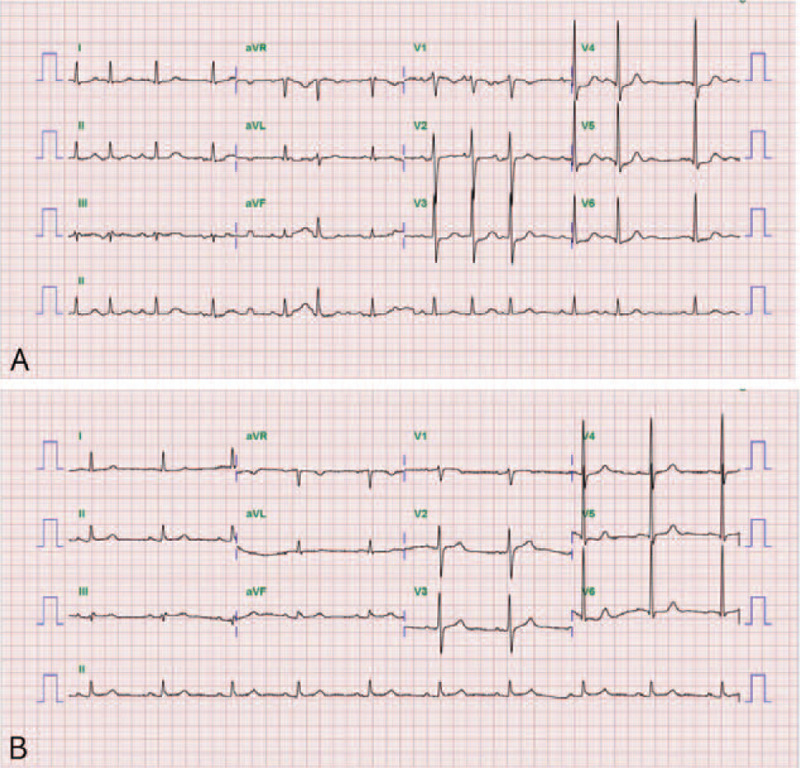
Resting electrocardiographs: (A) the day after surgery and (B) preoperatively.

**Figure 3:**
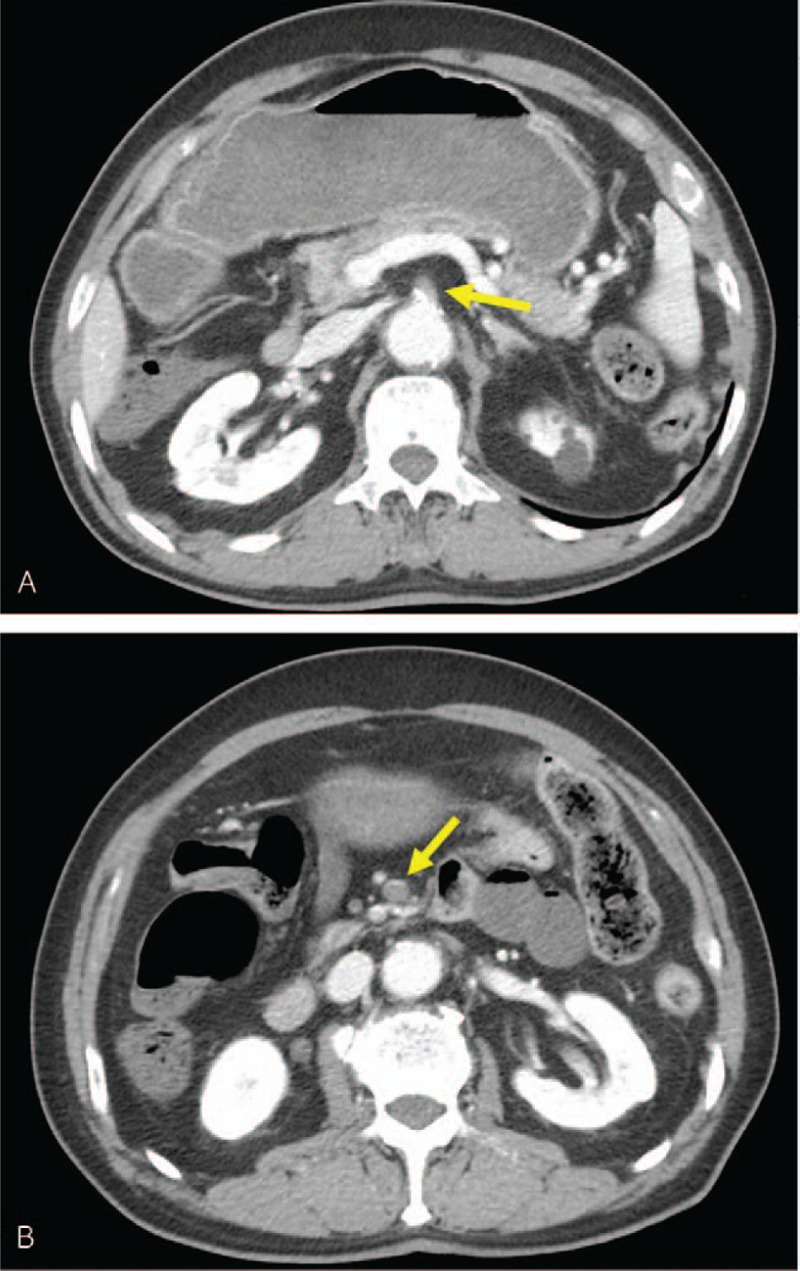
(A) and (B) Abdominal computed tomography scan showing occlusion of the superior mesenteric artery.

**Figure 4:**
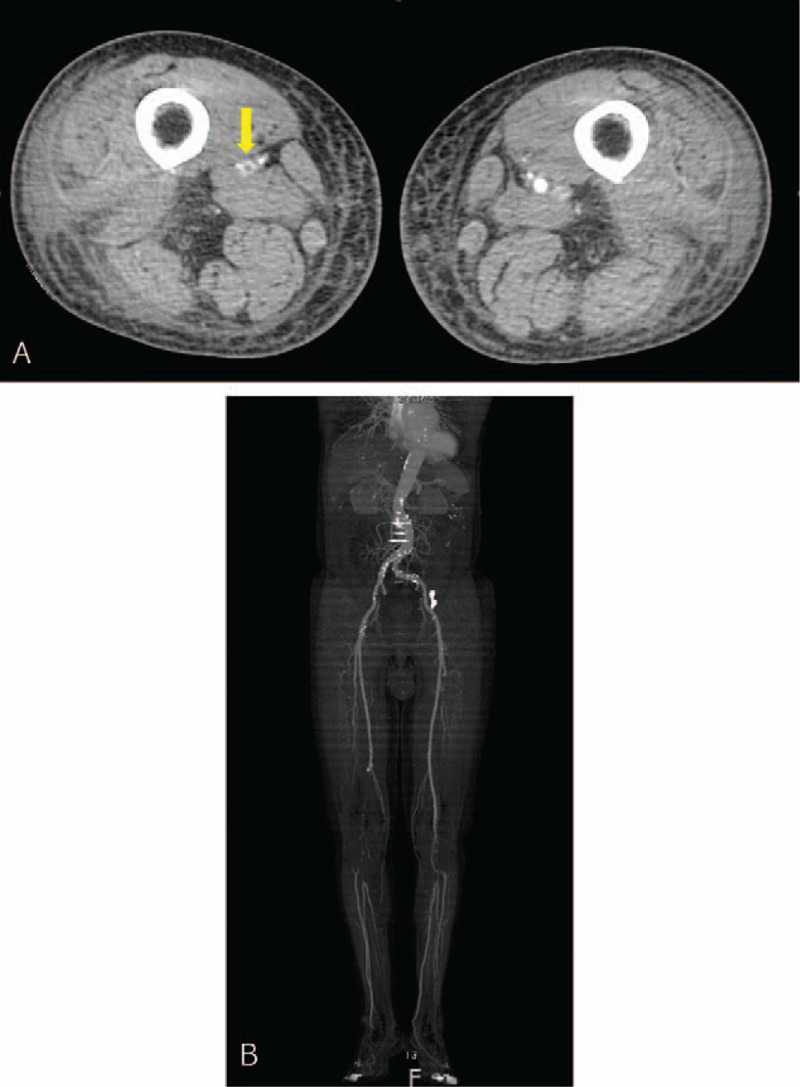
Photograph of the removed emboli.

**Figures 5:**
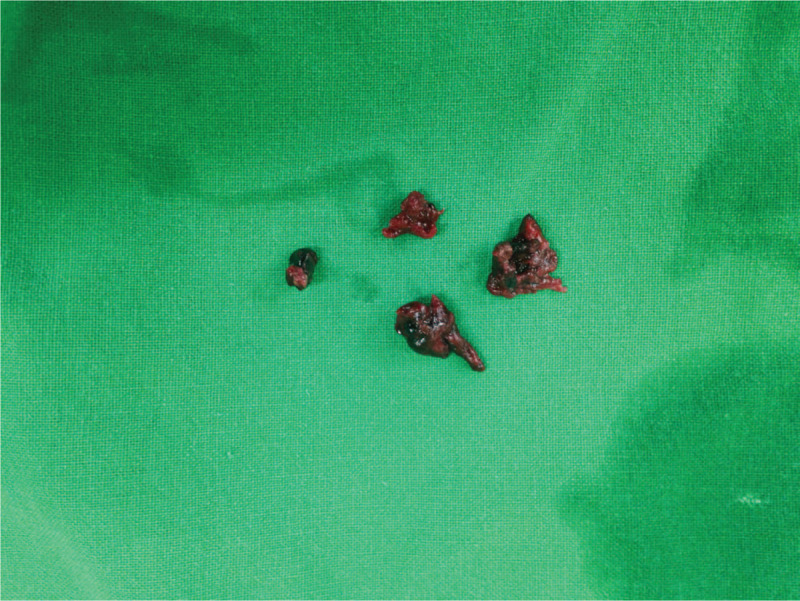
(A) & (B) Computed tomographic angiography scan of the low-extremity arterial system.

This report was approved by the Institutional Review Board of Bucheon St. Mary's hospital at the College of Medicine in the Catholic University of Korea in Korea (HC21ZISI0002). A 78-year-old male patient visited our outpatient department with a complaint of pain and edema in his right leg. He had no known comorbidities and no smoking history, and many varicose veins were observed on his right leg. Duplex ultrasonography (USG) revealed pathologic venous reflux at his right saphenofemoral junction, great and small saphenous veins, saphenopopliteal junction, and perforator veins, with no evidence of deep vein reflux and thrombosis (Fig. 1A and 1B). There were no abnormalities in the arterial system. As this patient had already received conservative management for superficial venous insufficiency for 3 months at the private clinic and showed no clinical improvement, we decided to operate on this patient. At this time, we had no equipment for venous ablation; therefore, we were obliged to perform saphenous and perforator vein ligation and stripping. We gave this patient and his family full details of the invasiveness and possible surgical complications, and they consented to this operation. A preoperative evaluation was performed for general anesthesia, and no abnormalities were found on electrocardiography (ECG), chest X-ray, or laboratory examinations. As scheduled, the patient received surgery with no intraoperative complications, and no medical events occurred on the day of the operation. The next morning after surgery, he complained of sudden epigastric pain, and his symptoms were aggravated despite analgesics and antispasmodics. First, ECG with cardiac markers was checked to rule out myocardial ischemia; however, we found a change on ECG, in which A-fib was newly discovered (Fig. 2A and 2B). Under the suspicion of acute mesenteric ischemia, an abdomen computed tomography (CT) scan was performed immediately, and it revealed embolic occlusion of the SMA (Fig. 3A and 3B). As the viability of the intestines was uncertain on CT scan, we decided to perform diagnostic laparotomy instead of angiographic intervention. The time to operation from the recognition of abdominal pain was 6 h. The peritoneal cavity was observed to be clean with no contamination, and the viability of the intestine was still grossly uncertain. We approached the root of the SMA and performed embolectomy through transverse arteriotomy using a balloon catheter until sufficient arterial back-flow from the distal portion of the SMA was identified (Fig. 4). Then, warm saline was added to the peritoneal cavity, and the movement, color, and tactility of the small and large intestines returned to normal. Finally, we performed rapid Doppler USG examination of the intestinal mesentery to check any remaining emboli. The operation took 2 h, and the patient was admitted to the surgical intensive care unit for postoperative care because he presented circulatory shock with anuria and metabolic acidosis after reperfusion. Under the suspicion of ischemic reperfusion injury, we started high-dose vasopressor treatment, fluid resuscitation, mechanical ventilation, and continuous renal replacement therapy to maintain adequate blood pressure and organ perfusion. Persistent A-fib was found on hemodynamic monitoring, and transthoracic echocardiography showed a normal size of each cardiac chamber with no valvular heart disease. We started anticoagulation with low molecular weight heparin on the 3^rd^ postoperative day (POD) to prevent systemic embolization. Although critical care was challenging in this patient, mechanical ventilation was able to be weaned off on the 7^th^ POD, and renal replacement therapy was weaned off on the 12^th^ POD. CT angiography was performed from the chest to the lower extremities on the 14^th^ POD, and it revealed complete occlusion of both popliteal arteries (Fig. 5A and 5B). We could not determine whether the occlusion was embolic or thrombotic; however, it did not change the treatment plan because arterial supplies were maintained through collateral vessels with no evidence of critical limb ischemia. After the patient became clinically stable, we performed endovascular thromboembolectomy and bypass surgery for low extremity arteries on the 34^th^ POD, and the patient responded well to postoperative treatment. He was discharged from the hospital on the 40^th^ POD with oral anticoagulant and antiplatelet agents and is now receiving regular check-ups for A-fib at the outpatient department of cardiology.

## 3 Discussion

In patients with known A-fib, elective noncardiac surgery can be delayed until the rate and rhythm become suitable for surgery, and anticoagulation treatment can be maintained perioperatively to prevent systemic embolization according to the risk-benefit strategy. However, some patients may present new-onset A-fib postoperatively, which is associated with systemic inflammation, infection, electrolyte abnormalities, increased adrenergic tone, hypoxia, hypervolemia, and anemia.^[5,6]^ We found that postoperative new-onset A-fib might be classified into two groups: one is truly new in patients with normal cardiac function, and the other is unveiled after surgery in patients who already have asymptomatic A-fib. It would be difficult to fully determine a patient‘s cardiac function preoperatively because the preoperative work-up usually depends on history taking and physical examination, especially in minor surgery. The American College of Cardiology and American Heart Association Task Force has recommended that preoperative resting ECG is reasonable only in patients with known coronary artery disease, significant arrhythmia, pulmonary vascular disease, or other significant structural heart disease.^[7]^ Moreover, paroxysmal A-fib might not be detected on preoperative resting ECG, and in cases with clinically asymptomatic postoperative A-fib, a diagnosis cannot be made unless the patient presents A-fib-related symptoms or signs, such as palpitations, dyspnea, lightheadedness, angina, and syncope, because such patients are usually not monitored postoperatively. Therefore, we suggest that the actual incidence of postoperative new-onset A-fib might be higher than that reported. Notably, A-fib following noncardiac surgery can increase mortality, length of hospital stay, and medical cost,^[1]^ with a similar risk of thromboembolic complications compared to that with nonvalvular A-fib.^[4]^ The goal of treatment for postoperative A-fib is to ensure hemodynamic stability with a rate- or rhythm-control strategy and to perform optimal anticoagulant treatment in high-risk patient groups.

To date, there is no evidence that varicose vein ligation and stripping might cause postoperative A-fib or have a higher complication rate than minimally invasive surgery, such as sclerotherapy or laser ablation.^[8,9]^ In a study of 973 limbs, there was no perioperative mortality, with a 2.8% incidence of wound complications, a 6.6% incidence of minor neurological disturbance, a 0.5% incidence of deep vein thrombosis, one case of pulmonary embolism, and one case of foot drop.^[9]^ Therefore, our case seems to be caused by a series of coincidences. This patient had few contributing factors for postoperative A-fib, which include male sex, prior history of A-fib, elevated heart rate, chronic kidney disease, sepsis, valvular heart disease, hypertension, increased preoperative B-type natriuretic peptide, and increased age.^[2,6,10,11]^ Moreover, the risk of embolization in this case was considered to be 0.6% (age ≥75 years = 1 point) according to CHA2DS2-VASc risk stratification scores,^[12]^ and the proportion of SMA occlusion is known to be 5% among total peripheral arterial embolic occlusions.^[13]^ In cases with acute mesenteric ischemia, the decision regarding treatment options should be made according to the viability of the involved intestine. Emergent laparotomy is indicated if the patient shows signs of advanced ischemia, such as peritonitis, sepsis, and pneumatosis intestinalis.^[14]^ Hemodynamically stable patients without clinical or radiologic signs of advanced intestinal ischemia may be candidates for an endovascular approach. However, there is no consensus regarding treatment options for patients in the gray zone. We went straightforwardly to laparotomy because we could not confirm the viability of the intestine on CT scan, and the preparation of the surgical team was faster than that of the interventional team.

On the basis of our experiences with this case, we suggest the following in a certain group of asymptomatic patients receiving surgery with a low risk of MACE:

1. preoperative resting ECG seems to be helpful;
2. postoperative telemetry might be required during the immediate postoperative period; and
3. atypical postoperative abdominal pain unresponsive to conservative treatment should be considered a surgical emergency requiring prompt evaluation and treatment that includes a high level of clinical suspicion for acute mesenteric ischemia. Future studies are required to investigate the topics arising from this report.

## Author contributions

**Conceptualization:** Jinbeom Cho, Dosang Lee.

**Supervision:** Dosang Lee.

**Writing – original draft:** Jinbeom Cho.

**Writing – review & editing:** Dosang Lee.
